# The Role of Interferons in Inflammation and Inflammasome Activation

**DOI:** 10.3389/fimmu.2017.00873

**Published:** 2017-07-25

**Authors:** Nataša Kopitar-Jerala

**Affiliations:** ^1^Department of Biochemistry, Molecular and Structural Biology, Jožef Stefan Institute, Ljubljana, Slovenia

**Keywords:** caspase-1, caspase-11, cyclic GMP-AMP synthase, guanylate-binding protein, interferon, inflammasome, macrophages, pyropotosis

## Abstract

Inflammation is an essential physiological process, which enables survival during infection and maintains tissue homeostasis. Interferons (IFNs) and pro- and anti-inflammatory cytokines are crucial for appropriate response to pathogens, damaged cells, or irritants in inflammatory response. The inflammasom is multiprotein complex, which initiates cleavage of pro-inflammatory cytokines IL-1β and IL-18 into active forms. In addition, inflammasomes initiate pyroptotic cell death. In the present review, I summarize and analyze recent findings regarding the cross talk of IFNs and inflammasomes.

## Introduction

Inflammation is a complex immune response to response to pathogens, damaged cells, or irritants and enables survival during infection or injury and maintains tissue homeostasis (1). In response to an infection, a cascade of signals leads to the recruitment of inflammatory cells (neutrophils and macrophages), which produce cytokines and chemokines (2). The sustained robust inflammation may lead to serious disorders due to the overproduction of inflammatory cytokines and tissue damage (2). However, cytokine secretion from neutrophils and macrophages is tightly regulated on the transcriptional level, and several pro-inflammatory cytokines also have posttranscriptional level of regulation (3). A typical inflammatory response consists of four components: inflammatory inducers, the sensors that detect them, the inflammatory mediators induced by the sensors, and the target tissues that are affected by the inflammatory mediators (1, 3). The innate immune response is involved in various inflammatory processes and has a particularly important role in bacterial and viral infections. Interferons (IFNs) and inflammatory cytokines are crucial molecules in this process, influencing cellular, tissue, and global physiological functions. Immune cells (macrophages, dendritic cells) recognize pathogen-associated molecular patterns (PAMPs) and endogenous danger-associated molecular patterns (DAMPs) (4, 5). Bacterial and viral PAMPs are detected by pattern recognition receptors (PRRs), which are also able to recognize DAMPs—endogenous molecules, released by dying or damaged cells (5–7). PRRs have distinct subcellular localization: toll-like receptors (TLRs) and C-type lectin receptors are transmembrane proteins found in the plasma membrane and endosomes, where they can survey PAMPs and DAMPs in the extracellular milieu. Intracellular PRRs are the retinoic acid-inducible gene I (RIG-I)-like receptor, the AIM2-like receptor (ALR), and the nucleotide-binding domain and leucine-rich repeat-containing (NLR) proteins (8). In addition, PRRs that sense cytosolic DNA and trigger the production of type I interferon were described (9). In this review, we discuss recent advances in understanding the role of IFNs in inflammatory response and inflammasome activation.

## Interferons

Interferons were first described as an antiviral factor that interferes with viral replication in mammalian cells (10). They are secreted from infected cells and activate innate immune response that promotes not only cytokine production but also natural killer cell functions and antigen presentation (11, 12). On the basis of the structural homology and the specific receptor they associate with, three classes of IFNs have been described (Type I, II, and III) (12). Type-I IFN family includes numerous IFN-α variants (13 in human and 14 in mouse), a single IFN-β; in addition, several other IFNs were reported (IFN-ε, -k, -ω, and -δ) (11, 13). IFN-γ, is the sole type II interferon, is structurally different from the type I and III IFNs, and signals through a different receptor: the IFN-γ receptor (3). IFN-γ can potentiate pro-inflammatory signaling by priming macrophages for antimicrobial actions, since it induces nitric oxide (NO) production and inhibit NLRP3 inflammasome activation (14, 15).

Type I-IFN expression is induced by activation of PRRs and by cytokines (9, 16). While, several different cell types express IFN-β, IFN-α is secreted only by hematopoietic cells, predominately plasmacytoid dendritic cells (17). Type I-IFNs are protective in acute viral infections; however, in bacterial infections, they could have either protective or deleterious roles (18). Type I-IFNs are induced by ssRNA, dsRNA, and cytosolic DNA from viruses or bacteria (19, 20). Type-I and type-II IFNs were reported to promote the expression of over 2,000 IFN-stimulated genes (ISGs), and the ISGs-induced proteins were demonstrated to act by enhancing pathogen detection and restrict the replication of pathogens (21). Several environmental factors, as well as host and pathogen factors, regulate responses of cells to IFN signaling (11).

Toll-like receptors are a family of 13 receptors known as PRRs and play a key role in the innate and adaptive immune response (22). Viral nucleic acids are recognized by endosomal TLR-3 (double-stranded RNA), TLR-7, -8 (single-stranded RNA), and TLR-9 (unmethylated CpG DNA) (4, 19). While TLR7 and TLR9 are expressed in B cells, macrophages, and DCs, TLR8 is expressed in macrophages and DCs In addition, TLR3 is broadly expressed also in non-hematopoietic cells, in humans. Triggering of PRR results in signaling pathways that activate gene transcription by nuclear factor (NF)-κB, as well as interferon regulatory factors (IRFs) and leads to production of type I IFNs and cytokines and chemokines (4, 23, 24). Endosomal TLR3 signals solely *via* the adaptor TIR domain-containing adaptor inducing IFN-β (TRIF), while TLR7, 8, and 9 depend on myeloid differentiation factor-88. Both pathways subsequently activate the IκB kinase (IKK) complex leading to nuclear translocation of the transcription factor NF-κB to upregulate the expression of inflammatory cytokines and chemokines (25). IRF transcription factors, crucial for the induction of type I IFNs (IFN-α and IFN-β), are also activated by endosomal TLRs. Signaling of TLR receptors and their adaptors result in transcription factors IRF3 and IRF7 activation, while IRF3 is expressed in many different cell types, plasmacytoid dendritic cells are the only cell type that constitutively express IRF7 (11). PRRs also induce activation of pro inflammatory caspases, leading to production of processed mature cytokines. Recently, also epigenetic mechanism that determines cell type-specific differences in IFN and IFN-stimulated gene (ISG) expression in response to exogenous signals was described (26).

Cytosolic DNA sensor proteins include cyclic GMP-AMP synthase (cGAS) (27) and ALR inflammasomes: Aim-2 and IFN-γ-inducible protein 16 (IFI16). Both Aim2 and IFI16 contain HIN200 domain that bind directly to DNA and a pyrin domain (28–30). Moreover, an endoplasmic reticulum-associated molecule referred to as stimulator of interferon genes (STING) was reported to control a signaling pathway important for the detection of cytosolic DNA and type I IFN expression (31, 32). Microbial RNAs are recognized by melanoma differentiation-associated gene 5 and (RIG-I), both of which are expressed in macrophages and non-hematopoietic cells (4, 19). Downstream signaling pathways are transmitted by mitochondrial antiviral mitochondrial antiviral signaling (MAVS), also known as IFN-β promoter stimulator-1 (IPS-1)/virus-induced signaling adaptor (VISA)/Cardif, a transmembrane protein on mitochondria (33). Recently, several excellent reviews describe the mechanism of nucleic acid sensing and signaling in the cytosol (34–36).

Interferon (IFN)-α and IFN-β bind to IFN-α receptor (IFNAR), a heterodimeric transmembrane receptor, which consist of two subunits: IFNAR1 and IFNAR2. Type I IFN-induced canonical signaling pathway IFNAR engagement activated the receptor-associated protein tyrosine kinases Janus kinase 1 (JAK1) and tyrosine kinase 2, which in turn phosphorylated the signal transducer and activator of transcription 1 (STAT1) and STAT2 (37). The activated STAT1 and STAT2 dimerize and rapidly translocate to the nucleus, where they together with IFN-regulatory factor 9 form a trimolecular complex called IFN-stimulated gene factor 3 (ISGF3) (11). ISGF3 binds to DNA sequences, which are known as IFN-stimulated response elements and directly activating the transcription of ISGs. Within a period of hours, however, the signal decays and the STATs are exported back to the cytoplasm for the next round of signaling (38, 39). Interestingly, the affinity of the IFNAR receptor varies between the different type I IFN ligands, due to the activation of different regulatory elements (40). However, the other cytokines activate STAT homodimers that recognize different gamma-activated sequence. Therefore, canonical type I IFN signaling induces a distinct subset of several hundred ISRE-driven ISGs. Cellular responses to IFNAR ligation vary during the course of an immune response and are cell type-and context-dependent (41).

Several different mechanisms were described that suppress type I IFN-mediated responses: downregulation of IFNAR expression on cell surface, induction of negative regulators like ubiquitin carboxy-terminal hydrolase 18 (USP18), and suppressor of cytokine signaling (SOCS). SOCS proteins compete with STATs for binding to IFNAR, while USP18 displaces JAK1 from IFNAR2 (42, 43). In addition, type I IFN responses are regulated by miRNAs (44, 45). During PRR and inflammatory signaling, miR-155 is highly induced (46, 47). It was reported that miR-155 suppressed the expression of IFNAR–JAK–STAT pathway in CD8^+^ T cells and the consequence of this suppression was enhanced CD8^+^ T cell responses to viral and bacterial pathogens (47).

Type-I and type-II IFNs are known to promote the expression of over 2,000 ISGs, and the products of ISGs have been shown to act by enhancing pathogen detection and innate immune signaling or restricting intracellular replication of viruses, bacteria, and parasites (21). Protein modification by the ubiquitin-like modifier interferon (IFN)-stimulated gene 15 (ISG15) is strongly induced by type I IFNs and represents one of the major antiviral IFN effector systems (48, 49). Conjugation of ISG15 to its substrates is counteracted by the activity of ubiquitin-specific protease 18 (USP18/UBP43) (50).

Another important group of proteins is superfamily of IFN-induced GTPases. Based on biochemical and structural studies, IFN-induced GTPases are grouped into four families of IFN-inducible, dynamin-like GTPases: the myxovirus resistance proteins (Mx), the immunity-related GTPases, the guanylate-binding proteins (GBPs), and the very large IFN-inducible GTPases (51). IFN-induced GTPases are transcribed in response to type-I, type-II, and type-III IFNs, while the Mx proteins are expressed only in response to type-I and type-III IFNs. TNF-α signaling was proposed to act as an alternative induction route for the GTPase; therefore, IFNs are not the only factors acting as GTPase inducers (52, 53). However, type-II IFNs and type-I IFNs are the strongest inducers, while TNFα and LPS are relatively weak stimuli.

## Inflammasomes

Activation of the inflammasome is a key event in inflammatory immune response. The inflammasomes are cytosolic multiprotein complexes that are composed of an inflammasome-initiating sensor, apoptosis-associated speck-like protein containing a CARD (ASC) acts as an adaptor protein and the protease-caspase-1. Inflammasome-initiating sensors include members of the NLRs the pyrin and HIN domain-containing (also known as PYHIN, Aim 2-like receptors, or ALRs; e.g., Aim2), or the TRIM (e.g., pyrin) family (54). Complex assembly leads to caspase-1-dependent cleavage of cytokines pro-interleukin 1β (pro-IL-1β) and pro-IL-18 into secreted mature forms (55–57). In addition, inflammasomes initiate pyroptotic cell death (52, 57, 58). Pyroptosis involves cell swelling, membrane rupture, and release of the cytoplasmic content into the extracellular space (58–60). Pyroptotic cell death is induced by caspase-1 or mouse caspase-11 (human caspase-4/5) cleavage gasdermin D (GSDMD), a pore-forming protein that normally exists in the auto inhibited state (58, 61, 62). Interestingly, since mature IL-1β lacks target sequences, secretion may require pyroptosis of the macrophages (60). However, other mechanisms of IL-1β secretion might also exist, human monocytes were reported to release IL-1β without pyroptosis (63).

Recently, several excellent reviews described mechanism of inflammasome activation (52, 56, 64, 65). Several NLR family members have been described as components of inflammasomes: Nlrp1b inflammasome (66, 67), Naip-Nlrc4 inflammasome (68, 69), the Nlrp6 inflammasome (70), the Nlrp12 inflammasome, the Aim2 inflammasome (28, 71), the RIG-I inflammasome (72), and the IFI16 inflammasome (73). Particularly, the activation of Nlrp3 inflamamsome is well characterized (55, 64, 74, 75). Since it responds to variety of stimuli, many different mechanisms of its activation have been proposed, including the release of oxidized mitochondrial DNA, production of reactive oxygen species and mitochondrial dysfunction, lysosomal destabilization, changes in intracellular calcium levels, the formation of large non-specific membrane. The Nlrp3 inflammasome activation in macrophages requires 2 steps: the first, priming step is provided by TLR signaling that upregulates NLPR3 and pro-IL-1β gene expression. This process is tightly controlled by signals culminating in the activation of NF-κB (76). Moreover, Nlrp3 activation can be regulated through direct posttranslational modifications, such as ubiquitination (77). Recently, several independent studies reported non-canonical inflammasome activation (78–80). While canonical inflammasome activation results in caspase-1 cleavage and activation, the activation of a non-canonical inflammasome results in activation of procaspase-11 (56). The mouse caspase-11 has high similarities to caspase-1 and is orthologous to human caspases-4 and -5 (81, 82). Both caspase-1 and caspase-4/11 could induce pyroptosis, while only caspase-1 processes proforms of IL-1β and IL-18 into secreted mature forms (78, 83). Only caspase-11-deficient mice, but not caspase-1-deficient mice were partially protected from septic death (78, 84). Recent reports showed that caspase-11 was involved in the response to cytosolic LPS, independently of TLR4 and was integral to the pathology of LPS-mediated endotoxic shock in mice (61). Moreover, it was shown that human caspase-4 and caspase-5 and mouse caspase-11 bound directly to LPS in the cytosol (85). With the difference to canonical inflammasome activation were the receptor (Nlrp3) and ASC form a scaffold on which caspase-1 can oligomerize, in non-canonical infalmmasome activation, caspase-11 oligomerization occurs directly upon binding to LPS (85). Human caspase-4/5/or mouse caspase-11 cleave GSDMD, a pore-forming protein that normally exists in the auto inhibited state (58, 61, 62). Furthermore, GSDMD N-terminal domain was found to associate with membranes, including the plasma membrane (86–89). It was reported that canonical Nlrp3 inflammasome activation downstream of caspase-4 and caspase-11 activation was dependent on potassium efflux (90–92). Yang et al. reported that cytosolic LPS stimulation induced caspase-11-dependent cleavage of the pannexin-1 channel followed up by potassium efflux and ATP release (92).

AIM2-like receptor inflammasomes are another class of inflammasomes that function to induce caspase-1 activation and IL-1β cytokine maturation. However, unlike NLR inflammasomes, ALR inflammasomes directly bind their ligand, dsDNA (28–30). While IFI16 recognizes dsDNA in the cytosol and nucleus, while Alm2 is localized only in the cytosol (93). In addition, IFI16 could induce type I IFN expression (30).

Inhibition of inflammasome activation by decoy proteins uses proteins structurally related to components of inflammasome and competing for the same adaptors. The CARD-only proteins and PYD-only proteins (POPs) function as endogenous dominant negative proteins that modulate the activity of inflammasomes and protect from excessive inflammation (94, 95). The genes encoding these decoy proteins, POPs, are located on the same chromosome, in the proximity of genes that encode their ligands: the gene encoding POP1 is located on human chromosome 16 next to the gene encoding ASC (96). POP3 has significant sequence similarity to the PYRIN domain of AIM2 (its target protein), encoded by a neighboring gene (97). Recently, it was demonstrated that POP2 not only prevented inflammasome assembly by binding to ASC but also impaired macrophage priming by inhibiting the activation of non-canonical IKK ε and IκBα (98).

## Cross Talk of IFNs and Inflammasomes

**Figure 1:**
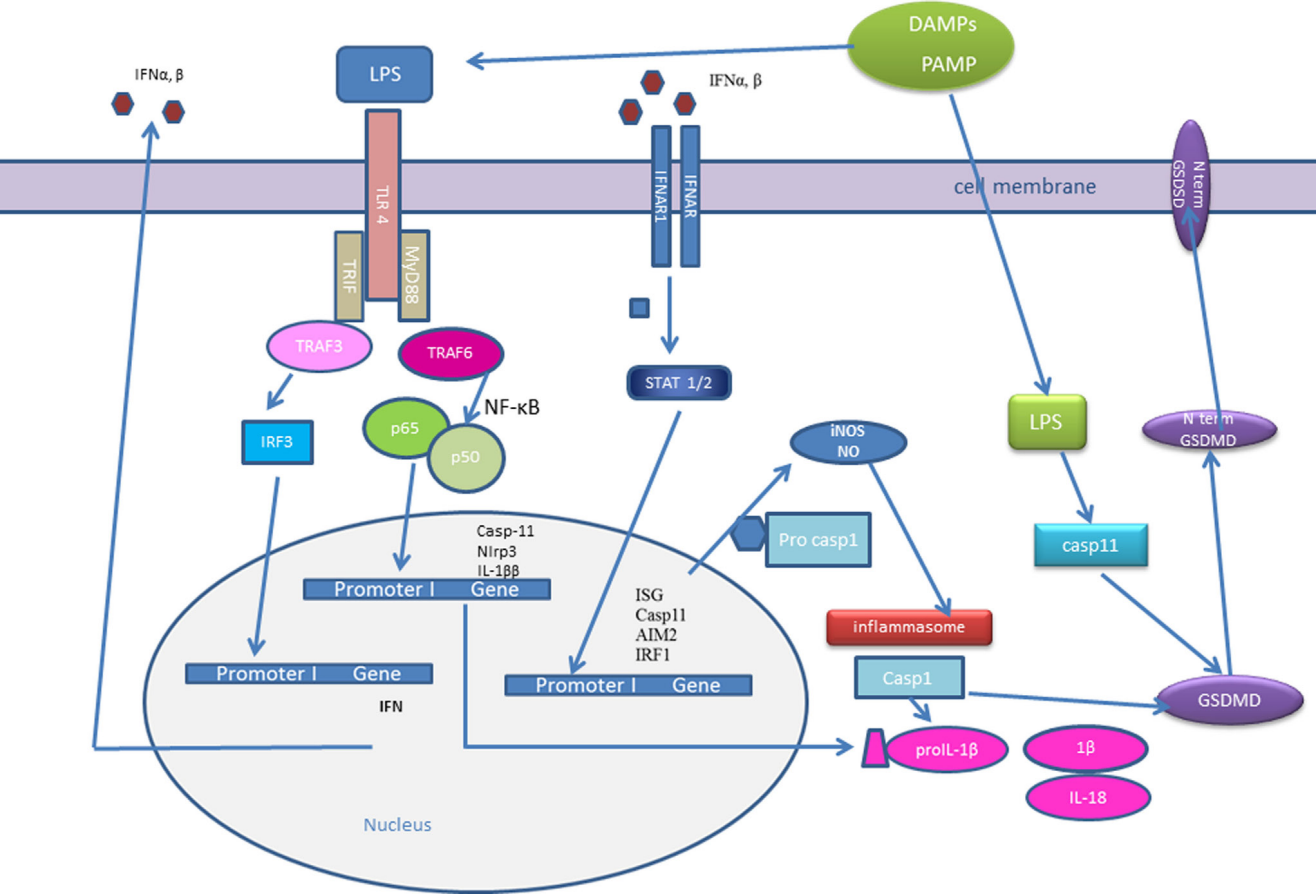
Type I interferons (IFNs) and inflammasome activation. Initial pathogen-associated molecular patterns (PAMPs) recognition by pattern recognition receptors induces IFN-β expression. IFNs could signal in an autocrine or paracrine manner and trigger expression of IFN-stimulated genes (ISGs): interferon regulatory factor (IRF)1, AIM2, caspase-11. Caspase-11 recognizes cytosolic LPS and induces IL-1β processing in an Nlrp3-dependent manner and triggers pyroptosis through gasdermin D (GSDMD) cleavage. Active caspase-1 and caspase-11 cleave GSDMD and the released gasdermin-N domain binds to phosphoinositides in the plasma membrane, oligomerizes to generate membrane pores, and initiates cell death-pyroptosis. IRF induce the expression of guanylate-binding proteins (GBPs), which target vacuolar and cytosolic bacteria, compromise the integrity of bacterial cells, and expose PAMPs like LPS and dsDNA to cytosolic sensors, caspase-11, and AIM2. IFN signaling triggers the expression of inducible nitric oxide synthase (iNOS), which upregulates cellular nitric oxide (NO) levels leading to NLRP3 S-nitrosylation.

Interferons could contribute to inflammasome activation through several different mechanisms (Figure 1). It was reported that type I IFNs are required for the caspase-11 expression, which contributes to activation of non-canonical inflammasome (79). Several recent studies have shown that IFN-inducible endogenous proteins could act also as negative regulators and thus inhibit inflammasome activation (97, 99). Among others, interferon-inducible GBPs not only mediate host resistance to pathogens but also promote inflammasome activation in bacterial infections (100, 101). Also, small proteins that are composed of either a CARD or a PYD only, emerged as important inflammasome regulators (94, 95). It was demonstrated that POP3, which is induced by type I IFNs, interacted with the PYD domain of AIM2 and competed with ASC to inhibit AIM2 inflammasome activation in response to dsDNA, mouse CMV, and modified vaccinia virus Ankara infection (97). Silencing of POP3 in human macrophages enhanced DNA and DNA virus-induced ALR inflammasome formation and hence the maturation and release of IL-1β and IL-18 (97). Not only POPs but also metabolites like 25-hydroxycholesterol, an oxysterol and is derived from cholesterol, suppress inflammasome activation (99). At least in macrophages, IFN-β strongly induced cholesterol 25-hydroxylase, the enzyme that transforms cholesterol into 25-hydroxycholesterol (102, 103). Work of Reboldi et al. showed that 25-hydroxycholesterol inhibited not only pro-IL-1β gene transcription but also the inflammasome activation (99). The authors proposed that 25-hydroxycholesterol antagonized the sterol response element-binding protein processing (99). Moreover, cholesterol 25-hydroxylase-deficient mice showed increased sensitivity to LPS-induced septic shock (99).

Both type-I IFNs and IFN-γ could promote inducible nitric oxide synthase (iNOS), which increases the amount of endogenous NO, expression in macrophages (15, 104). NO plays an important role in a defense against pathogens, it could be oxidized to reactive nitrogen oxide species, that *S*-nitrosate thiols in proteins (15, 104). Mishra et al. reported that NO inhibited NLRP3 oligomerization by means of direct S-nitrosylation of the NLRP3 protein, preventing full inflammasome assembly (15). Also study by Mao et al. demonstrated that NO prevented the activation of the NLRP3 inflammasome (14). In line with the above results, in iNOS-deficient macrophages, NLRP3 inflammasome activation was enhanced, iNOS-deficient mice had increased mortality from LPS-induced sepsis (14).

In addition, type I IFN signal *via* STAT1 decreased the activity of Nlrp3 inflammasome that induce caspase-1 to process the IL1-β precursor in response to a large variety of intracellular PAMPs (105). Different mechanisms could contribute to diminished IL1-β processing in IFN-stimulated cells. STAT1 target gene products directly repress NLRP3 inflammasome. Moreover, the IFN-I/STAT1 pathway increases IL-10 synthesis, IL-10-mediated STAT3 activation, and the suppression of IL1-β precursor synthesis by activated STAT3 (106). Guarda et al. showed that IL-1α and IL-1β were downregulated in mice pretreated with poly(I:C), a synthetic RNA analog that strongly induces type-I IFNs (106). In addition, they demonstrated that the recruitment of inflammatory cells (neutrophils and monocytes) into peritoneal cavity was significantly lower in poly(I:C) pretreated mice, than in control animals injected only with LPS. Moreover, they demonstrated that IFN-β suppress not only inflammasome activation and IL-1β secretion but also it rendered the mice more susceptible to *Candida albicans* infection (106).

Several recent studies reported cross talk between IFNs and inflammasome activation in bacterial infections (79, 100, 101, 107, 108). An early study showed that caspase-11 gene expression in response to LPS and IFN-γ was dependent on NF-κB and STAT-1 signaling (109). Rathinam et al. demonstrated that transcriptional induction of caspase-11 by IFN-β signaling was enough to induce both its expression and auto activation (79). Gurung et al. reported that TLR4–TRIF–IFNβ-induced caspase-11 synthesis is crucial for non-canonical Nlrp3 inflammasome activation in macrophages infected with enteric pathogens *Escherichia coli* and *Citrobacter rodentium* (110). IFN-γ could also upregulate caspase-11 expression. Aachoui et al. showed that caspase-1 activity is required upstream of caspase-11 to control infection by cytosolic bacterium *Burkholderia thailandensis*. Caspase-1-activated IL-18, which further induced IFN-γ to prime caspase-11 and rapidly clear *B. thailandensis* infection. Whereas IFN-γ was essential, endogenous type I IFNs were insufficient to prime caspase-11 and cleared *B. thailandensis* (111). Oficjalska et al. reported that IFN-γ-dependent, type I IFN–TRIF-independent signaling pathway was required for *in vivo* caspase-11 production in intestinal epithelial cells during DSS-induced colitis (112). However, LPS-stimulated macrophages from TRIF-deficient mice had impaired caspase-11 expression, implying a context-dependent role for type I or II IFN in the regulation of caspase-11 activity (79, 112). In addition, IFN-γ induced upregulation of Nlrp3, ASC, and procaspase-1 expression (100, 113, 114). IFN-γ enhanced Aim2-induced IL-1β release or Nlrp3-dependent pro-IL-18 cleavage during HSV-1 and *Chlamydia muridarum* infections (115, 116).

Upon bacterial infection, IFN-inducible GTPases—GBPs target vacuolar and cytosolic bacteria and compromise the integrity of bacterial cells, thus exposing the microbial ligands LPS and DNA to cytosolic sensors caspase-11 and Aim2 (100, 101). GBPs have also been shown to regulate the entry of LPS into the cytosol by, as yet, poorly defined mechanisms (100). Significant reduction in NLRP3 inflammasome activation was reported in GBP5-deficient macrophages infected with *S. typhimurium* or treated with potassium efflux agonists (117). However, studies on different mouse strain of GBP5*-*deficient mice could not confirm the initial results (108, 114). Despite the uncertainty surrounding the role of GBP5 in Nlrp3 inflammasome activation, studies using mice lacking the entire cluster of GBP genes on chromosome 3, have firmly confirmed a functional link between GBPs and the activation of the canonical NLRP3 and AIM2 inflammasomes, as well as the non-canonical caspase-11 inflammasomes. Recently, GBP2 emerged as a critical activator of AIM2 and caspase-11 inflammasomes (100, 101). GBP2 is induced by type I or II IFNs and exposes Gram-negative bacteria-derived LPS to caspase-11 (114). In addition, it was shown that IFN-β boosts canonical AIM2-dependent IL-1β secretion to *Francisella tularenis* or *Listeria monocytogenes* (71, 118) and helps to control caspase-11-dependent pyroptosis by Gram-negative bacteria (79, 107).

**Figure 2:**
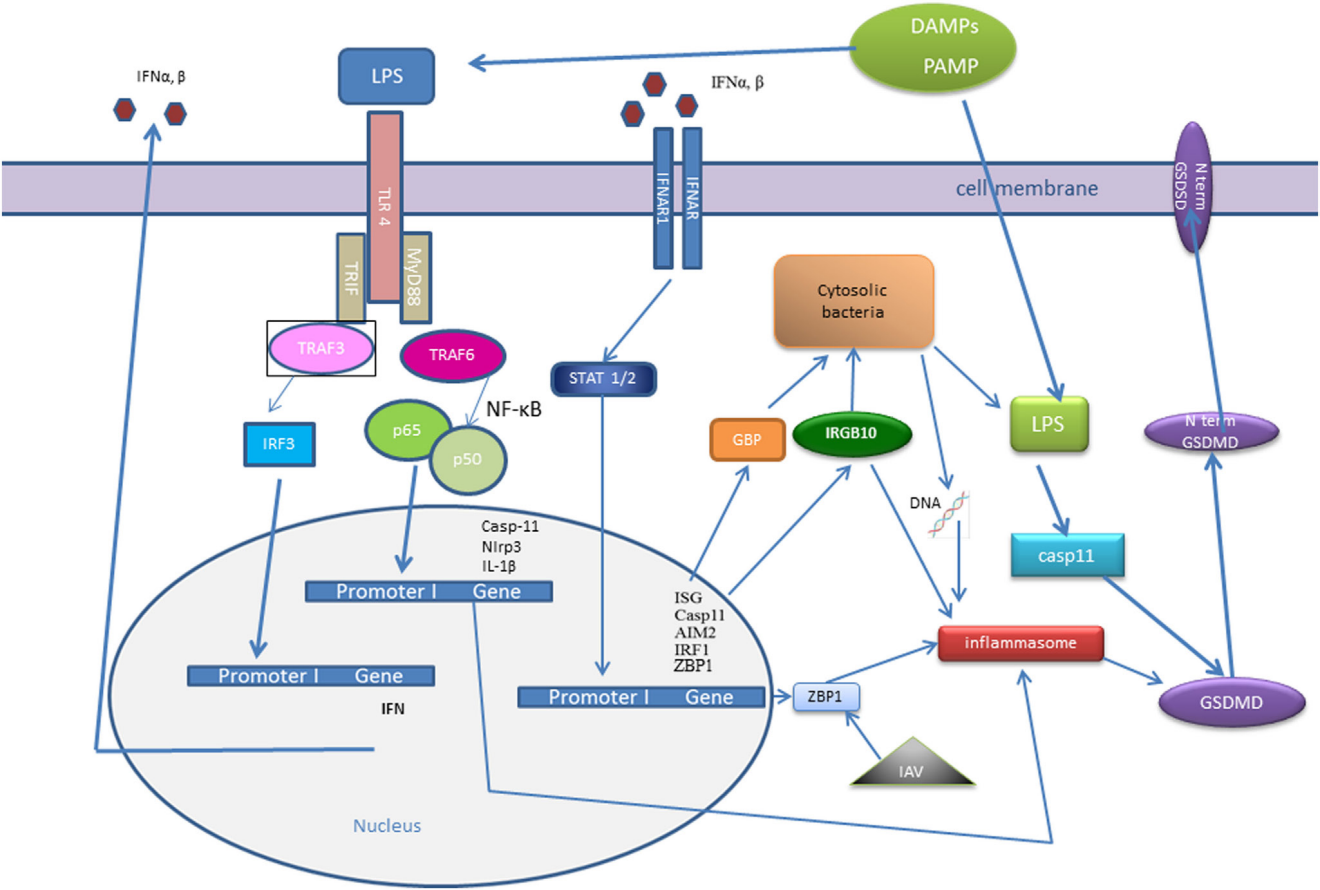
Interferon (IFN) signaling influence recognition of intracellular pathogens—cytosolic bacteria and influenza A virus (IAV). IFNs signaling trigger the transcription factor interferon regulatory factor (IRF)1, which promotes expression of guanylate-binding proteins (GBPs) and interferon response gene B10 (IRGB10). IRGB10, together with GBPs permeabilizes the membrane of Gram-negative bacteria, an action that results in release of bacterial DNA and LPS. Bacterial cytosolic DNA is sensed by Aim2 inflammasome and LPS directly interacts with caspase-11. Type I IFN signaling mediates upregulation of interferon-inducible protein Z-DNA-binding protein 1 (ZBP1), which recognizes the IAV proteins and triggers NLRP3 inflammasome activation, as well as induction of apoptosis, necroptosis, and pyroptosis in IAV-infected cells.

Type I-IFN signaling is also essential in response to *Francisella novicida* infection (101, 119). *F. novicida* DNA is detected by DNA sensor cGAS, which induced STING-dependent production of type I-IFNs (71). Type I-IFNs in act *via* the transcription factor IRF1, which regulates expression of GBPs and IRG (108, 120) (Figure 2). Interferon response gene B10 together with GBP2, GBP5 work synergistically to rupture *F. novicida* that have entered the cytoplasm, and their action result in the exposure of *F. novicida* DNA for sensing by DNA sensor AIM2 (52, 101, 114).

Another IFN-inducible protein, Z-DNA-binding protein 1 (ZBP1), also known as DNA-dependent activator of IFN-regulatory factors (DAI), has been known as a cytosolic DNA sensor for almost a decade (121). However, a recent work demonstrated that ZBP1 could sense the RNA virus, influenza A virus (IAV) proteins: nucleoprotein and polymerase subunit 1. Kuriakose showed that in IAV-infected cells, ZBP1 regulated NLRP3 inflammasome activation, as well as induction of apoptosis, necroptosis, and pyroptosis (122) (Figure 2). ZBP1-deficient mice were protected from mortality during IAV infection, due to reduced inflammatory response (122).

## Concluding Remarks

I have summarized considerable, but by no means all evidence documenting the role of IFNs in inflammasome activation and inflammation. Several recent studies reported the essential role of type I IFNs in non-canonical Nlrp3 inflammasome activation and pyroptosis. Different levels of regulation are involved in the cross talk of IFNs in inflammasome. Not only type I-IFNs but also IFN-γ influence caspase-11 expression and consequently pyroptosis. Dysregulated type I-IFN production could lead to a cell death. However, a recent study reported that in the absence of active proapoptotic caspases-3 and -7, mitochondrial outer membrane permeabilization by Bax and Bak resulted in the expression of type I-IFNs. The process was mediated by mitochondrial DNA-dependent activation of the cGAS/STING (123). Particularly, the role of STAT and other protein modification in IFN signaling pathways could give us important insight into the regulatory mechanisms. IFN-induced GBPs were reported to have an important role in caspase-11 activation and pyroptotic cell death. How does the polymorphisms of GBPs influence inflammasome activation and inflammation is yet to be determined. Future research should explore the detailed molecular mechanisms that are responsible for type I IFN-dependent cell death and inflammasome activation in inflammatory response. Moreover, recently, several studies determined the role of cytokines in metabolic reprograming and inflammasome activation (124). The role cross talk of IFNs, inflammasomes, and metabolism could be a future frontier for the cutting edge research. Identification of the factors involved in inflammasome regulation and signaling will lead to the identification of novel targets for therapeutic intervention.

## Author Contributions

The author confirms being the sole contributor of this work and approved it for publication.

## Conflict of Interest Statement

The author declares that the research was conducted in the absence of any commercial or financial relationships that could be construed as a potential conflict of interest.
